# Granuloma Annulare of the Palms in a Patient on Ribociclib for Metastatic Breast Cancer

**DOI:** 10.7759/cureus.73159

**Published:** 2024-11-06

**Authors:** Aliya Rogers, Joanna Jaros

**Affiliations:** 1 Dermatology, Ascension Resurrection Medical Center, Chicago, USA; 2 Dermatology, North Branch Dermatology, Chicago, USA

**Keywords:** breast cancer, granuloma annulare, oncodermatology, paraneoplastic granuloma annulare, ribociclib

## Abstract

Granuloma annulare (GA) is a benign inflammatory skin condition that most commonly presents on the dorsal surfaces of the hands and feet. The etiology of GA is unknown; however, it has been associated with multiple triggers, including malignancy and targeted cancer therapy drugs. This case report describes a 66-year-old female with metastatic breast cancer on ribociclib who presented with painful, erythematous papules on the palmar surfaces of the hands. Histopathology confirmed a diagnosis of GA, indicating a rare presentation of GA on the palms.

## Introduction

Granuloma annulare (GA) is a benign skin condition with multiple clinical presentations. The most common presentation of GA is localized GA, which presents as pink to red non-scaly annular plaques classically involving the dorsal surfaces of the hands and feet [1]. Other clinical presentations include generalized GA and subcutaneous GA, with more rare presentations including perforating GA, patch GA, and palmoplantar GA [1]. Histologically, the principal finding in all subtypes of GA includes mucin with a palisading or interstitial pattern of granulomatous inflammation [1]. The etiology of GA is unknown; however, there have been reported associations of GA with malignancy, including lymphoma, leukemia, and solid organ malignancies [2]. There have also been reports of multiple medications that can trigger GA, such as tumor necrosis factor-alpha inhibitors, allopurinol, topiramate, gold therapy, and targeted cancer therapy drugs [2-4]. We present the case of a 66-year-old female with metastatic breast cancer on ribociclib who presented with painful, erythematous papules on the palmar surfaces of the hands identified by histopathology as GA.

## Case presentation

**Figure 1:**
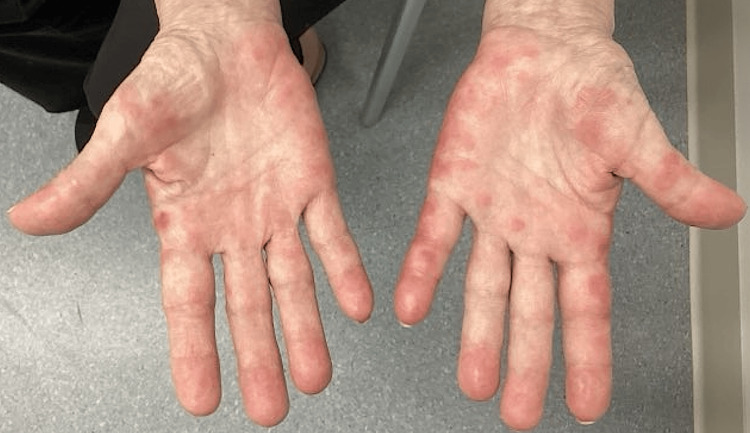
Tender, round, erythematous, and edematous papules, with some coalescing into plaques, distributed on the bilateral palms

A 66-year-old female presented to the dermatology clinic for evaluation of a painful rash on her hands for five days. She had a history of recurrent metastatic breast cancer diagnosed five months prior and started taking ribociclib (cyclin-dependent kinase 4 and 6 inhibitors) four months ago. A physical exam revealed tender, round, erythematous, and edematous papules, with some coalescing into plaques, distributed on the bilateral palms (Figure 1). A punch biopsy was performed on the left hypothenar eminence.

**Figure 2:**
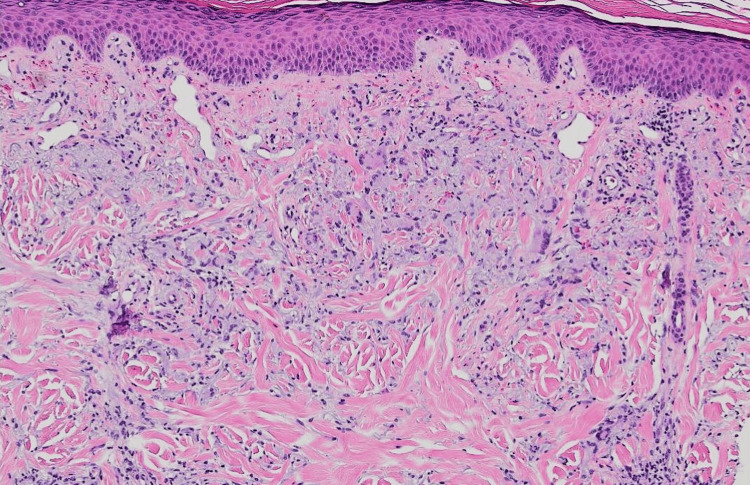
Altered collagen in the dermis with mucin deposition and a palisade of histiocytes and interstitial histiocytic inflammation (H&E, original magnification ×100)

**Figure 3:**
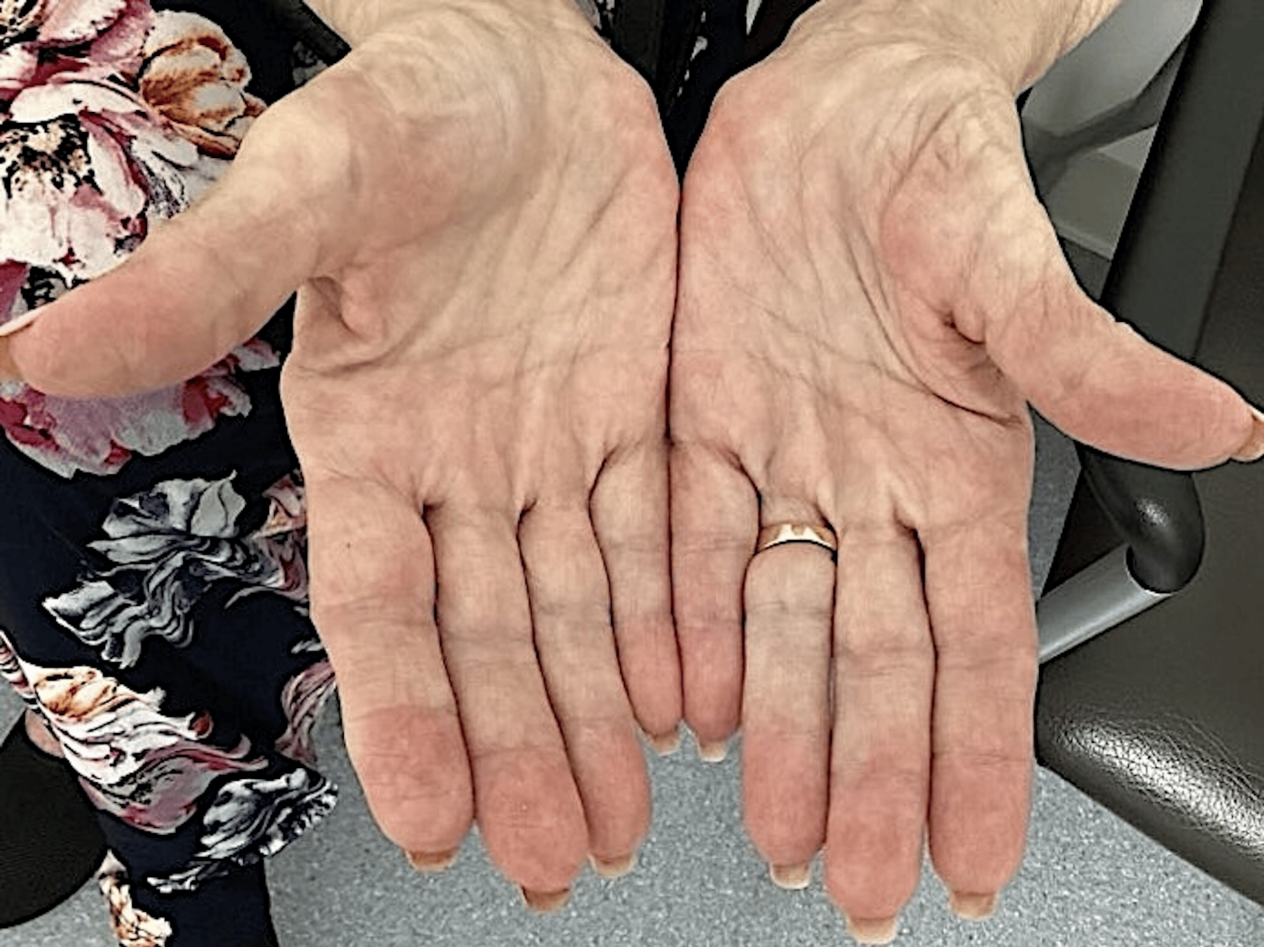
Resolution of GA with post-inflammatory erythema after applying clobetasol cream twice daily for five weeks GA, granuloma annulare

Histopathology of the lesion showed focal zones of altered collagen in the dermis and a wispy blue material consistent with mucin. There was a palisade of plump histiocytes around the zones and interstitial histiocytic inflammation (Figure 2). This was most consistent with a diagnosis of GA. The patient was advised to apply clobetasol cream twice daily to the affected areas. At her follow-up one month later, the rash had resolved with post-inflammatory erythema (Figure 3). She has had no recurrences to date, and she continues to be on ribociclib.

## Discussion

Our patient presented with GA on the palms, which is a rare presentation of GA. In a study of seven patients with GA on the palms, it was found that the most common clinical symptom was pain, and there were various clinical morphologies, including pseudovesicles, erythematous papules, and keratotic papules [5]. The authors commented that diagnosing GA on the palms is often challenging, with the diagnosis being initially missed in five of seven cases [5]. Therefore, for papular lesions on the palms, GA should be considered in the differential, and it is important to obtain a biopsy for a definitive diagnosis.

The etiology of GA is unknown and debated in the literature. Recent evidence suggests that GA may be triggered by a cell-mediated, hypersensitivity reaction where helper T-cells interact with macrophages to cause granulomatous inflammation [2]. This hypothesis could help explain the rare association of GA and malignancy. One proposed explanation of its pathogenesis is a tumor-related alteration of cell-mediated immunity [6]. In a case-control study of paraneoplastic GA in patients with solid organ malignancies, paraneoplastic GA was defined as GA occurring within six months of the diagnosis of malignancy and/or persistent GA that resolved with cancer treatment [6]. This study found that most cases of paraneoplastic GA occurred in patients with lung or breast cancer, were generalized and/or atypical in their presentations, and improved with treatment of the cancer [6]. In our patient’s case, it is possible that she had paraneoplastic GA, as it occurred within six months of her recurrence of metastatic breast cancer. However, our patient’s GA presented while she was receiving cancer treatment (ribociclib) and experiencing marked improvement in tumor load on imaging.

GA has also been found to be triggered by certain drugs, including targeted cancer therapy drugs. Two cases of GA were reported in patients receiving vemurafenib, a BRAF inhibitor, for metastatic malignant melanoma [3]. Another case of GA was reported in a patient receiving nivolumab, an immune checkpoint inhibitor, for lung cancer [4]. In our case, the patient was receiving ribociclib, a cyclin-dependent kinase 4 and 6 (CD4/6) inhibitor, when she developed GA. To our knowledge, in the literature, there is only one reported case of GA triggered by a CD4/6 inhibitor [7]. CD4/6 inhibitors have been found to decrease levels of regulatory T-cells and increase levels of CD4+ T-cells [8]. Therefore, this drug could have potentially triggered GA through the hypothesized cell-mediated hypersensitivity reaction.

There is a scarcity of evidence-based therapies for GA in the literature. A proposed treatment ladder based on a systematic review of GA treatment options includes topical or intralesional corticosteroids as first-line therapy, phototherapy or hydroxychloroquine as second-line therapy, and dapsone as third-line therapy [9]. There are reports of improvement after trauma to the lesions, including biopsy, cryotherapy, incision, and pricking [10], and, recently, there have been reports of oral JAK inhibitors as an effective treatment option [11]. There are no specific guidelines for the selection of treatment for GA; it is important to consider factors such as cost, availability, side effects, body surface area affected, and clinical efficacy when choosing a treatment option.

In our patient, the differential diagnosis included hand-foot skin reaction (HFSR), neutrophilic eccrine hidradenitis (NEH), Sweet syndrome, mycosis fungoides palmaris et plantaris (MFPP), and sarcoidosis, which were all ruled out based on histopathology. HFSR is a cutaneous adverse effect of kinase inhibitor therapy. It is characterized by sharply demarcated, erythematous, edematous, tender papules and plaques with blister and callus formation on palmoplantar surfaces [12]. Histopathological findings include epidermal keratinocyte apoptosis, keratinocyte vacuolar degeneration, and intraepidermal blister formation [12]. HFSR is associated with multikinase inhibitors [12], and ribociclib is a selective kinase inhibitor, making this diagnosis less likely.

NEH is a type of neutrophilic dermatosis most commonly occurring in patients with acute myeloid leukemia who are receiving chemotherapy; however, it has also been reported in patients with solid organ malignancies and patients receiving targeted cancer therapy [13]. NEH most commonly presents as asymptomatic or painful erythematous edematous papules or plaques [13]. It can occur on the palms, but the trunk is most often affected [13]. Diagnosis is based on histopathology, which shows eccrine glands with degenerative vacuolar changes [13].

Sweet syndrome is another type of neutrophilic dermatosis that classically presents as an abrupt onset of painful erythematous plaques or nodules and can also be associated with malignancy [13]. There is a localized variant called neutrophilic dermatosis of the dorsal hands that can involve the palmar surfaces as well. Histologically, it is characterized by predominantly neutrophilic infiltrates in the dermis [13].

MFPP is a rare variant of cutaneous T-cell lymphoma that primarily involves the palms and soles and has various clinical presentations that can mimic multiple inflammatory palmoplantar dermatoses [14]. The histopathology of MFPP demonstrates infiltrates of a clonal population of T-cells [14].

Sarcoidosis is a chronic, multisystem inflammatory disorder characterized by histopathology revealing non-caseating granulomas. It is known as a “great imitator” due to its various morphological presentations [15]. A case in the literature identified painful palmoplantar nodules as the initial manifestation of sarcoidosis, similar to our patient’s clinical presentation [16].

## Conclusions

This case highlights a rare presentation of GA on the palms, treated with topical clobetasol, in a 66-year-old female on ribociclib for metastatic breast cancer. The rash occurred five months after the diagnosis of metastatic breast cancer and four months after starting ribociclib. Possible triggers for GA in this case include malignancy or an adverse reaction from ribociclib therapy. Although it rarely presents on palmar surfaces, it is important to consider GA in the differential diagnosis of a painful papular rash on the palms. In these cases, obtaining a biopsy is critical for confirming a diagnosis.
